# Unusual lung involvements of invasive mucinous adenocarcinoma with chylothorax

**DOI:** 10.1111/1759-7714.13665

**Published:** 2020-09-18

**Authors:** Ayako Aoki, Yu Hara, Koji Okudela, Yoshihiro Ishikawa, Kosei Doshita, Hisashi Hashimoto, Kentaro Nakashima, Nobuyuki Horita, Nobuaki Kobayashi, Takeshi Kaneko

**Affiliations:** ^1^ Department of Pulmonology Yokohama City University Graduate School of Medicine Yokohama Japan; ^2^ Department of Pathology Yokohama City University Graduate School of Medicine Yokohama Japan; ^3^ Department of Thoracic Surgery Yokohama City University Hospital Yokohama Japan

**Keywords:** Chylothorax, computed tomography, invasive mucinous adenocarcinoma

## Abstract

A 77‐year‐old man who had a persistent productive cough for one month was admitted to our hospital. Chest computed tomography (CT) revealed subpleural nodular opacities, irregular pleural thickening with bilateral basal predominance, and a small right pleural effusion. Aspirated fluid was exudative and had the appearance of chylothorax without malignant cells. Surgical lung biopsy specimen showed focal proliferation of neoplastic epithelial cells with lepidic‐predominant pattern and abundant mucus in the alveolar spaces, consistent with invasive mucinous adenocarcinoma (IMA). The results of PD‐L1 expression and the EGFR, ALK, ROS1, and BRAF mutation status analyzed by next generation sequencer were all negative. IMA should be considered in the differential diagnosis of subpleural micronodular opacities accompanied by pleural effusion (chylothorax) on chest CT.

**Key points:**

**Significant findings of the study**

This case showed subpleural micronodular opacities and chylothorax as unusual chest computed tomography (CT) patterns for invasive mucinous adenocarcinoma (IMA).

**What this study adds**

Invasive mucinous adenocarcinoma (IMA) should be considered in the differential diagnosis of subpleural micronodular opacities accompanied by pleural effusion on chest CT.

## Graphical abstract

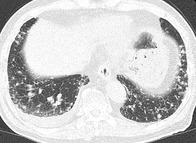

This case of invasive mucinous adenocarcinoma (IMA) showed subpleural micronodular opacities and chylothorax as unusual chest computed tomography (CT) patterns for IMA. IMA should be considered in the differential diagnosis of subpleural micronodular opacities accompanied by pleural effusion on chest CT.

A 77‐year‐old man with a history of a persistent productive cough for one month was admitted to our hospital. Chest computed tomography (CT) revealed subpleural nodular opacities, irregular pleural thickening with bilateral basal predominance, and a small right pleural effusion (Fig 1). The differential diagnosis included diffuse pan‐bronchiolitis, mycobacterium infection, pulmonary sarcoidosis, and lymphoma; however, there was no increase in blood angiotensin‐converting enzyme or carcinoma‐specific tumor markers including carcinoembryonic antigen, squamous cell carcinoma antigen, cytokeratin 19 fragment, neuron specific enolase, sialyl lewis X‐i antigen, or soluble interleukin‐2 receptor; and no evidence of specific pathogens or malignant cells in the sputum. After administration of macrolide and new quinolone antibiotics, the pleural effusion worsened slightly and lung involvement became more apparent. Aspirated fluid was exudative (protein; 2.8 g/dl, lactate dehydrogenase; 331 mg/dL), with a total cell count of 775/μL and lymphocyte predominance. It also had the appearance of chylothorax, with elevated triglyceride levels (160 mg/dL [pleural effusion] vs. 128 mg/dL [blood]) and without malignant cells. Although 18F‐fluorodeoxyglucose positron emission tomography‐computed tomography (18F‐PET‐CT) revealed no remarkable FDG uptake, surgical lung biopsy was performed to obtain a definitive diagnosis of lung involvement. The histological specimen showed focal proliferation of neoplastic epithelial cells with lepidic‐predominant pattern and abundant mucus in the alveolar spaces (Fig 2). Immunohistochemical results were CK7 (+), TTF‐1 (−), CK20 (−), HNF‐1α (−), and Ki‐67 (≥30%), consistent with invasive mucinous adenocarcinoma (IMA). The results of PD‐L1 expression and the EGFR, ALK, ROS1, and BRAF mutation status analyzed by next‐generation sequencing (NGS) were all negative.

**Figure 1:**
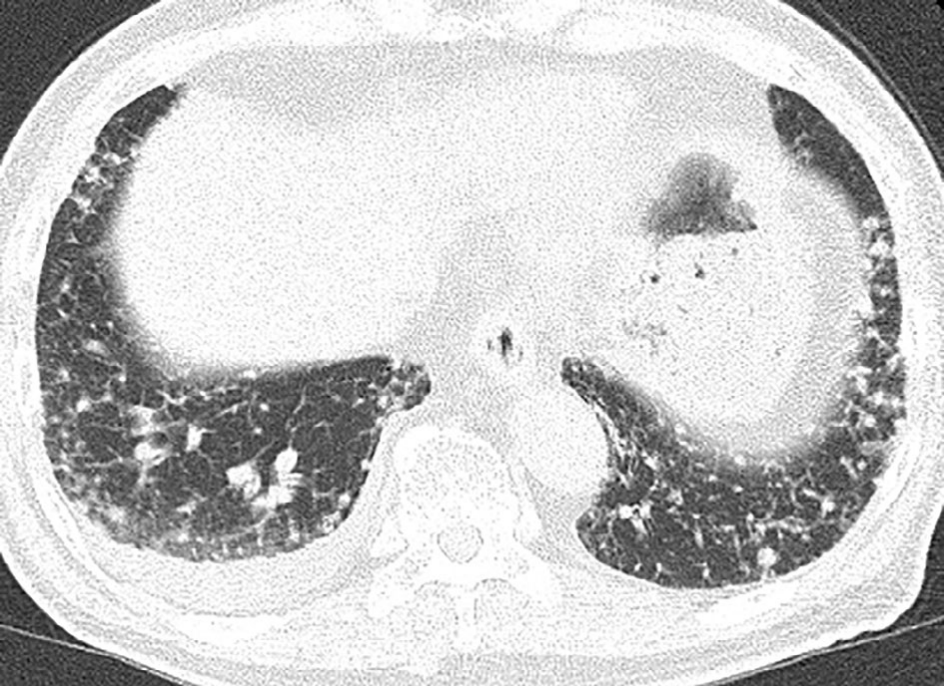
Chest computed tomography. Subpleural nodular opacities and irregular pleural thickening are seen with bilateral basal predominance. A small right‐sided pleural effusion is also observed.

**Figure 2:**
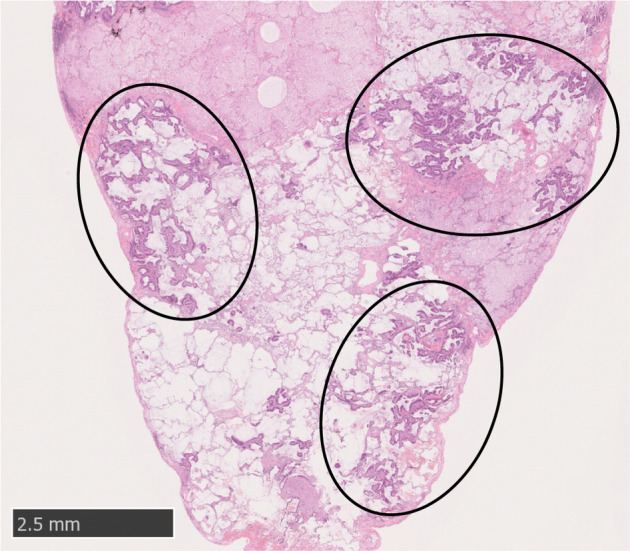
Surgical lung biopsy specimen. Histologically, the specimen showed focal proliferation of neoplastic epithelial cells with lepidic‐predominant pattern (black circles) and abundant mucus in the alveolar spaces.

Typical CT findings of IMA of the lung are a mass‐like opacity or consolidation mimicking lobar pneumonia.^1^, ^2^, ^3^ However, this case showed a very rare CT pattern of subpleural micronodular opacities that was difficult to identify as lung carcinoma. Moreover, this case was accompanied by chylothorax due coincidentally to tumor progression. Therefore, IMA should be considered in the differential diagnosis of subpleural micronodular opacities accompanied by pleural effusion on chest CT.

## Disclosure

None of the authors have any real or perceived conflicts of interest to declare in relation to this manuscript.
